# Distinct Functional Traits of Lactobacilli from Women with Asymptomatic Bacterial Vaginosis and Normal Microbiota

**DOI:** 10.3390/microorganisms8121949

**Published:** 2020-12-09

**Authors:** Rinku Pramanick, Clara Aranha

**Affiliations:** Department of Molecular Immunology and Microbiology, ICMR-National Institute for Research in Reproductive Health, Parel, Mumbai 400102, India; rinku.pramanick@gmail.com

**Keywords:** *Lactobacillus*, lactic acid, vaginal microbiota, bacterial vaginosis, asymptomatic BV, probiotic properties

## Abstract

Asymptomatic bacterial vaginosis (BV) in reproductive-age women has serious obstetric and gynecological consequences. Despite its high incidence, the behavior of vaginal lactobacilli in asymptomatic BV is unknown. We analyzed the functional properties of previously isolated vaginal lactobacilli from asymptomatic women with normal, intermediate, and BV microbiota. Lactic acid and antimicrobial activity against seven urogenital pathogens were evaluated from lactobacilli cell-free culture supernatants (CFCs) (*n* = 207) after 48 h incubation in MRS. Lactobacilli isolates were used to evaluate H_2_O_2_, autoaggregation and coaggregation with *C. albicans.* Lactobacilli from normal microbiota produced more d-lactate than lactobacilli from intermediate and asymptomatic BV (*p* = 0.007). *L. plantarum, L. fermentum* and *L. reuteri* produced greater d-lactate whereas *L. rhamnosus, L. crispatus*, *L. johnsonii* were greater producers of l-lactate. Interspecies positive correlation was observed in the lactic acid contents of CFCs. Distribution of H_2_O_2_-producing lactobacilli did not vary significantly among the groups. When lactic acid isomers were considered, species from intermediate and BV microbiota clustered together with each other and distinctly from species of normal microbiota. Broad-spectrum antagonism (≥90% inhibition) against *E. coli, C. albicans, S. aureus, P. aeruginosa, G. vaginalis, N. gonorrhoeae, S. agalactiae* were displayed by 46.86% (97) of isolates. Our study highlights the differential functional properties of vaginal lactobacilli from women with normal microbiota and asymptomatic BV.

## 1. Introduction

Vaginal microbiota are one of the critical features in maintaining vaginal homeostasis and providing protection against urogenital infections. Dysbiosis of the vaginal microbial composition could lead to bacterial vaginosis (BV) and other associated infections such as vulvovaginal candidiasis, trichomoniasis, and sexually transmitted infections (STIs) [1,2,3]. Bacterial vaginosis is the most common reproductive tract infection in women which could be symptomatic (with symptoms) or asymptomatic (without manifestation of clinical symptoms) [4]). Apart from symptomatic cases, incidences of asymptomatic BV can be 14.5% to as high as 84% [4,5,6]. Both symptomatic and asymptomatic BV has been associated with severe obstetrics’ and gynecological consequences [7,8,9,10]. Asymptomatic BV has been identified as a risk factor for late miscarriage and preterm delivery [8], and persistent infection of HPV [11]. Despite the increased risk of other infections, reproductive morbidity, and complications, asymptomatic BV is understudied.

We previously reported that vaginal microbiota of asymptomatic BV are different from healthy microbiota. Predominant *Lactobacillus* communities in eubiosis were *L. iners, L. crispatus, L. reuteri, L. jensenii*, and *L. gasseri* while women with asymptomatic BV harbored *L. iners, L. rhamnosus, L. reuteri, L. salivarius* and *L. johnsonii* [6]. Lactobacilli in the vaginal ecosystem play a protective role by limiting the growth, proliferation, and colonization of pathogens. These beneficial microbes contribute to the control of infections by producing antimicrobial compounds which include organic acids [12,13], hydrogen peroxide [14], bacteriocins and biosurfactants [15]. The presence of H_2_O_2_-producing lactobacilli strains during pregnancy has been associated with reduced risk of BV and adverse gynecological consequences [16]. Besides, lactobacilli can compete with pathogens for adherence to vaginal epithelial cells and prevent their colonization [17]. The mechanism of competitive exclusion could be due to the coaggregation of lactobacilli with the pathogenic microorganisms thus hindering the adherence and colonization of pathogens on the vaginal epithelium [18]. Another exclusion mechanism could be due to autoaggregation where *Lactobacillus* can form multi-cellular aggregates with bacteria from the same species and their adherence to epithelial cells and surfaces of mucus creates a barrier for pathogens [19].

Thus, *Lactobacillus* plays a protective role in the vaginal microenvironment, and reduction in their abundances and diversity leads to dysbiosis [6]. However, it is uncertain if the probiotic properties of lactobacilli, inhabiting the vaginal tract, vary during eubiosis and dysbiosis. So, we aimed to study the probiotic functional properties of lactobacilli isolated from normal and BV microbiota of asymptomatic women.

## 2. Material and Methods

### 2.1. Isolation of Lactobacilli

In this study, 207 *Lactobacillus* isolates were previously recovered from 145 healthy premenopausal, regularly menstruating participants of reproductive age asymptomatic for any vaginal complaints [6]. The study had the approval of the human ethics review board at the institute (Protocol Number 215/2012, 14 September 2012) and the collaborating institute (Sheth G.S and KEM hospital) (Protocol No EC/GOV-5/ 2012, 8 July 2013). Classification of the vaginal samples was done according to Nugent scoring [20]. We adopted the standardized 0–10 point Nugent scoring system based on identifying proportions of three bacterial morphotypes on Gram-stained vaginal smears: large Gram-positive rods (*Lactobacillus* spp.), small Gram-negative or Gram-variable coccobacilli (*Gardnerella* and anaerobic spp.), and curved Gram-variable rods (*Mobiluncus* spp.). A score of 0–3 was considered normal, 4–6 was intermediate, and scores ≥7 indicated BV. The lactobacilli were isolated from 116 women with normal microbiota, 11 with intermediate microbiota, and 18 women with asymptomatic BV. All the *Lactobacillus* strains had been isolated as colonies on MRS (deMan Rogosa and Sharpe) (HiMedia, Mumbai, India) agar medium after incubation for 24 to 48 h in a candle jar. The isolates further grown in MRS broth (HiMedia, Mumbai, India) were stored as glycerol stocks (20%) at −80 °C. For the present study, glycerol stocks of the 207 lactobacilli isolates were streaked on MRS agar and incubated at 37 °C for 48 h in a candle jar. 

### 2.2. Preparation of Cell-Free Culture Supernatants

Colonies of lactobacilli obtained on MRS agar were inoculated in MRS broth followed by incubation at 37 °C for 48 h in a candle jar. About 100 µL of lactobacilli cultures was used to measure bacterial growth at 600 nm using Synergy H1 Hybrid Multi-mode reader (BioTek Instruments, Winooski, VT, USA). Cell-free culture supernatants (CFCs) were obtained by centrifuging the culture medium at 10,000× *g* for 10 min at 4 °C. Centrifuged CFCs were passed through a sterile Millex GS filter unit (0.22 µm) (Millipore, Darmstadt, Germany). The pH of CFCs was measured using HiIndicator^TM^ pH papers (Himedia, Mumbai, India).

### 2.3. Determination of Lactic Acid

Briefly, 100 µL of lactobacilli CFCs was used to quantify d-/l- and total lactic acid using the d-/l-Lactic Acid test kit (Megazyme, K-DLATE, Co. Wicklow, Ireland) according to the manufacturer’s instructions. The amount of d-lactate and l-lactate was calculated after taking the absorbance at 340 nm using a microplate reader (BioTek Instruments, Winooski, VT, USA).

### 2.4. Determination of Hydrogen Peroxide 

The *Lactobacillus* isolates were plated onto MRS agar containing horseradish peroxidase (HRP) (Sigma-Aldrich, St Louis, MO, USA) and 3,3′,5,5′-tetramethylbenzidine (TMB) (Sigma-Aldrich, St Louis, MO, USA) using the protocol mentioned elsewhere [21]. After incubation, colonies were exposed to ambient air. Colonies turning to blue color were considered H_2_O_2_ producers. Depending on the intensity of blue, isolates were graded semi-quantitatively as strong, moderate, and weak producers.

### 2.5. Semi-Quantitative Estimation Lactobacilli Agglutination with C. albicans

Slide agglutination of a clinical *C. albicans* with lactobacilli was carried out as per a previous protocol with slight modifications [22]. Briefly cells were washed and resuspended in sterile PBS to achieve 1.0 OD_600 nm_. About 20 μL of previously isolated vaginal *C. albicans* CA119 (Pramanick et al., 2019) suspension was added to 20 μL of *Lactobacillus* cell suspension, along with 5 μL of crystal violet. Visual and microscopic agglutination was observed after 10 min of incubation. 

### 2.6. Quantitative Evaluation of Coaggregation and Autoaggregation

Overnight cultures of lactobacilli and *C. albicans* CA119 grown in MRS and Sabourauds broth (HiMedia, Mumbai, India), respectively, were centrifuged at 10,000× *g* for 10 min, followed by washing the cell pellet twice with sterile PBS. After washing cells were resuspended in sterile PBS and adjusted to 0.1 OD_620 nm_. For coaggregation assay, 100 µL each of *Lactobacillus* and *C. albicans* cell suspensions were added on a 96-well microplate. For autoaggregation, only 200 µL of *Lactobacillus* was added to each well. After 3 h of incubation at 37 °C, 100 µL of the upper supernatant was removed and OD_620 nm_ was recorded.

Coaggregation and autoaggregation were calculated using the following equations:

% Coaggregation = [(A_0_ − A_s_)/A_0_] × 100. (1)

A_0_ refers to the initial OD_620 nm_ taken immediately after both the cultures were mixed. A_s_ refers to the OD_620_ of the supernatant after 3 h incubation.

% Autoaggregation = [(A_0_ − A_s_)/A_0_] × 100. (2)

A_0_ refers to the initial OD_620_, and A_s_ refers to the OD_620_ determined after 3 h [23].

### 2.7. Antagonistic Activity of Lactobacilli Metabolites against Pathogens

The inhibitory activity of lactobacilli CFCs were assessed against different urogenital pathogens, *Escherichia coli ATCC* 25922, *Candida albicans CA119* (clinical sample), *Staphylococcus aureus ATCC* 25923, *Neisseria gonorrhoeae ATCC* 43069 *and Pseudomonas aeruginosa ATCC* 27853, *Gardnerella vaginalis ATCC* 14019, *Streptococcus agalactiae ATCC* 12386.

*G. vaginalis*, *N. gonorrhoeae* and *S. agalactiae* were grown in Brain-heart infusion (BHI) broth (HiMedia, Mumbai, India). Mueller Hinton (MH) broth (HiMedia, Mumbai, India) was used to grow *E. coli*, *S. aureus*, and *P. aeruginosa* and Sabouraud’s broth (HiMedia, Mumbai, India) was used to culture *C. albicans*. The overnight grown cultures of pathogens were, washed and resuspended in phosphate-buffered saline (PBS) to obtain 0.1 OD_600 nm_ cell suspension. The pathogen cell suspensions were further diluted 1:100 in the respective growth medium to prepare the inoculum. In a 96-well microtiter plate containing 100 µL lactobacilli CFCs, 100 μL pathogen inoculum was added into each well. Media control with no microbial culture and a positive control containing pathogen inoculum and MRS broth were used as negative control and positive control for growth. CFCs of each pathogen were used to determine the effect of any nutrient exhaustion in the metabolites. The plates were incubated under anaerobic conditions at 37 °C for 48 h. The growth of the cultures was measured at OD_600 nm_ after 4 h, 18 h, 24 h, and 48 h incubation. Percent growth inhibition was calculated using the following formula,

% Growth = [(Positive control OD_600_ − Test well OD_600_)/Positive control OD_600_] * 100 (3)

% Inhibition = 100 − % Growth(4)

### 2.8. Statistical Analysis

Data were analyzed using the Graphpad Prism 8.0.1 software. Results are the mean of experimental readings taken at least in duplicates. Lactic acid concentrations are represented in mM. Readings are expressed as mean ± std.deviations. Data of qualitative variable H_2_O_2_ was analyzed using the chi-square test and, Fisher’s exact test. Other variables were analyzed using Kruskal–Wallis one-way analysis of variance followed by pairwise multiple comparisons using Dunn’s multiple comparison test. Spearman’s rank correlation was used to determine the relationship between the variables. Statistical significance was considered at *p* < 0.05.

## 3. Results

### 3.1. Lactobacillus Isolates Diversity

For this study, 207 lactobacilli from 145 reproductive age women with no vaginal complaints and infections were assessed. These samples were previously classified as normal microbiota (116), intermediate (11) and BV (18) (Pramanick et al., 2019). Of the 207 lactobacilli, 169 were recovered from normal microbiota, 15 from intermediate, and 23 from asymptomatic BV. These 207 lactobacilli comprised of 12 different *Lactobacillus* species that constitutes *L. reuteri* (42, 20.29%), *L. rhamnosus* (40, 19.32%), *L. gasseri* (22, 10.62%), *L. jensenii* (18, 8.69%), *L. crispatus* (14, 6.76%) and *L. salivarius* (14, 6.76%).

### 3.2. Acidification of Medium

**Figure 1:**
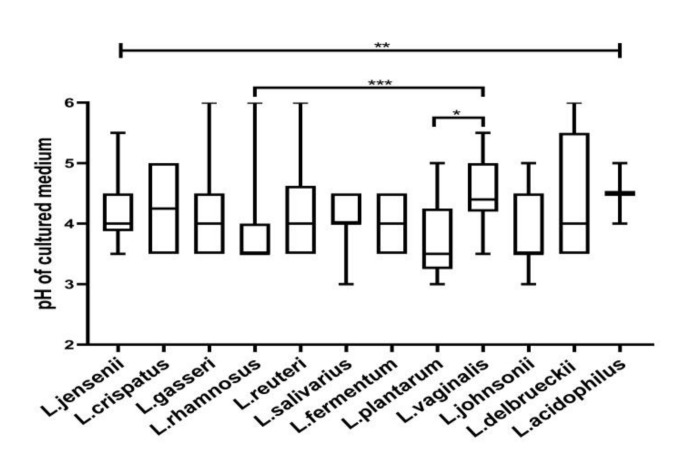
The acidity of CFCs (cell-free culture supernatants) was measured after the growth of different *Lactobacillus* species. The pH of the CFCs varied significantly among the species, as determined by the Kruskal–Wallis test. The topmost line indicates the Kruskal–Wallis test and downward pointing lines indicate Dunn’s multiple comparison test. * *p* < 0.05, ** *p* < 0.001, *** *p* < 0.001. Data are represented as a box plot wherein the box indicates the interquartile ranges, line within the bars represents the median and whiskers represent the minimum and the maximum values.

Lactobacilli in the vaginal milieu determine the vaginal pH. Herein, we evaluated the pH-buffering capacity of lactobacilli isolated from normal, intermediate, and BV microbiota in growth medium. The maximal OD600 nm of lactobacilli cultures in MRS ranged from 0.7 to 0.9. The CFCs of lactobacilli from normal, intermediate, and BV microbiota had an average pH of 4.17, 4.27, 3.96, respectively, with no significant difference (*p* = 0.4). The acidifying ability of *Lactobacillus* species differed significantly from each other (Figure 1). The average pH of *L. plantarum, L. fermentum, L. rhamnosus, L. johnsonii* CFCs were below 4. *L. acidophilus, L. delbrueckii*, and *L. vaginalis* isolates acidified the medium poorly. Acidifying potential of different isolates of the same species was similar regardless of vaginal microbiota (Figure S1).

### 3.3. Lactic Acid Quantification

**Figure 2:**
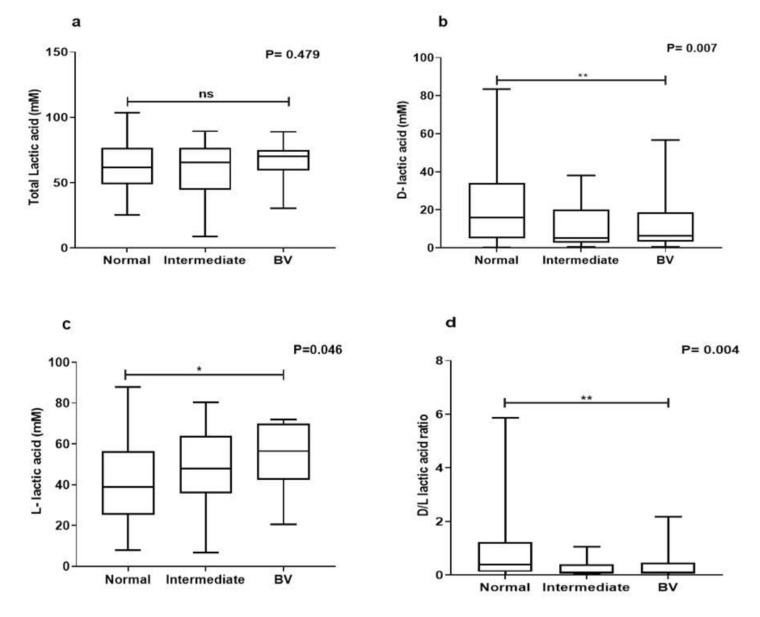
Lactic concentrations: (**a**) total lactic acid, (**b**) d-lactic acid, (**c**) l-lactic acid, (**d**) d-/l-lactic acid ratio of lactobacilli recovered from different vaginal microbiota. The amount of lactic acid isomers and d-/l-lactic acid ratio of lactobacilli metabolites differed significantly between the groups. Data are represented as mean ± SD. Ns = not significant and * *p* < 0.05, ** *p* < 0.01 indicating statistical significance after performing the Kruskal–Wallis test.

All *Lactobacillus* isolates produced lactic acid. Both isomers of lactic acid were detected in the metabolites of all *Lactobacillus* isolates. The mean lactic acid concentrations in metabolites of lactobacilli from normal microbiota (62.78 ± 16.98) did not differ significantly from intermediate (59.71 ± 24.74) and BV microbiota (66.74 ± 14.21) (*p* = 0.478) (Figure 2a). However, a higher amount of mean d-lactic acid was produced by lactobacilli from normal (21.34 ± 18.75) microbiota compared to that produced by lactobacilli from intermediate (11.42 ± 12.27) and BV microbiota (12.87 ± 15.27) (*p* = 0.007) (Figure 2b). l-lactic acid in metabolites of lactobacilli from normal microbiota (41.98 ± 20.13) was significantly less than intermediate (47.29 ± 21.84) and BV microbiota (52.33 ± 17.88) (*p* = 0.04) (Figure 2c). Moreover metabolites of lactobacilli from normal microbiota (0.77 ± 0.92) had a better d-/l- lactic acid ratio than those picked from intermediate (0.28 ± 0.32) and BV samples (0.39 ± 0.61) (*p* = 0.004) (Figure 2d).

### 3.4. Species-Specific Lactic Acid Production

Our studies showed significant difference in lactic acid isomer production from lactobacilli isolated from the three groups. Hence, we further investigated whether the production of lactic acid and its isomers could be attributed to a particular species. *L. plantarum* isolates produced the highest lactic acid in the medium (74.11 ± 15.72) followed by *L. johnsonii* (69.80 ± 18.39), *L. rhamnosus* (68.77 ± 17.26) and *L. fermentum* (67.58 ± 17.03). Furthermore *L. plantarum* (42.19 ± 23.45) *L. fermentum* (38.09 ± 21.71), *L. reuteri* (38.09 ± 21.71), produced higher d-lactic acid and *L. rhamnosus* (57.61 ± 18.49), *L. crispatus* (53.93 ± 18.84), *L. johnsonii* (47.33 ± 21.62) were higher producers of l-lactic acid. The average d-/l-lactic acid ratio of *L. plantarum* (1.70 ± 1.31), *L. fermentum* (1.35 ± 0.97), *L. delbreuckii* (1.13 ± 1.19) metabolites were higher, while metabolites of *L. rhamnosus* (0.20 ± 0.25), *L. crispatus* (0.29 ± 0.47), *L. gasseri* (0.41 ± 0.50) had the lowest average d/l ratios (Table S1).

**Figure 3:**
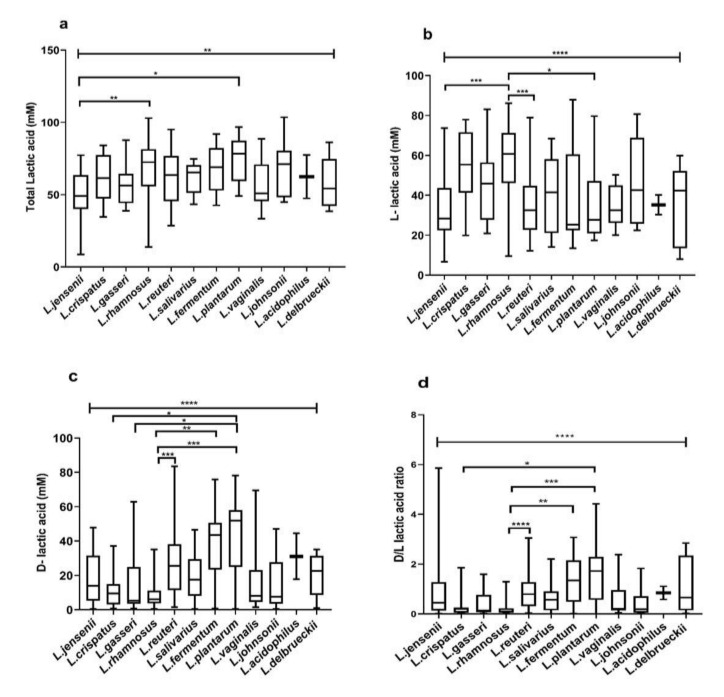
Lactic concentrations: (**a**) total lactic acid, (**b**) d-lactic acid, (**c**) l-lactic acid, (**d**) d-/l-lactic acid ratio of lactobacilli recovered from different vaginal microbiota. The amount of lactic acid isomers and d-/l-lactic acid ratio of lactobacilli metabolites differed significantly between the groups. * *p* < 0.05, ** *p* < 0.01, *** *p* < 0.001, **** *p* < 0.0001 indicating statistical significance after performing the Kruskal–Wallis test. The topmost line indicates the Kruskal–Wallis test and downward pointing lines indicate Dunn’s multiple comparison test. Data are represented as a box plot wherein the box indicates the interquartile ranges, line within the bars represents the median and whiskers represent the minimum and the maximum values.

The total lactic acid in metabolites of the different lactobacilli species differed significantly (*p* = 0.003) (Figure 3a). Metabolites of *L. plantarum* (*p* = 0.01) and *L. rhamnosus* (*p* = 0.006) contained a significantly higher amount of lactic acid than *L. jensenii* (Figure 3a).

Additionally, species-specific variations in l- lactic acid (*p* = <0.0001) (Figure 3b), d-lactic acid (*p* = <0.0001) (Figure 3c) and d-/l- lactic acid ratios were noted (*p* = <0.0001) (Figure 3d). The average d-lactic acid produced by *L. plantarum* was significantly higher than that produced by *L. crispatus* (*p* = 0.02), *L. gasseri* (*p* = 0.01)*, L. rhamnosus* (*p* = 0.0002). Likewise, *L. fermentum* (*p* = 0.002) and *L. reuteri* (*p* = 0.0008) isolates produced significantly higher d-lactic acid than *L. rhamnosus* (Figure 3b). l-lactic acid was significantly higher in CFCs of *L. rhamnosus* than *L. jensenii* (*p* = 0007), *L. reuteri* (*p* = 0.0002) *and L. plantarum* (*p* = 0.02) (Figure 3b). Metabolites of *L. reuteri* (*p* < 0.0001)*, L. fermentum* (*p* = 0.002)*, L. plantarum* (*p* = 0.0003) had a significantly greater d-/l-lactic acid ratios than *L. rhamnosus*. Likewise, metabolites of *L. plantarum* had higher d-/l- lactic acid ratio than *L. crispatus* (*p* = 0.03) (Figure 3d).

### 3.5. Intraspecies Comparison of Lactic Acid Production from Different Microbiota

**Figure 4:**
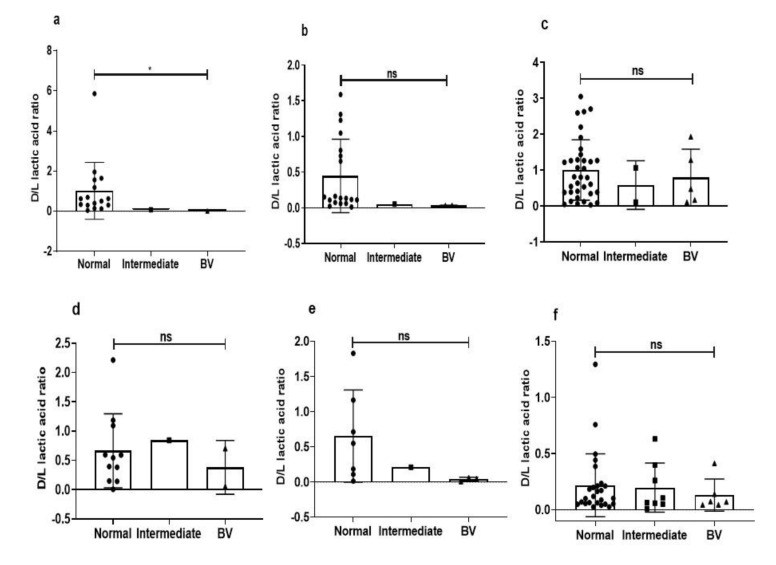
d-/l- lactic ratios of (**a**) *L. jensenii*, (**b**) *L. gasseri*, (**c**) *L. reuteri*, (**d**) *L. salivarius*, (**e**) *L. johnsonii*, (**f**) *L. rhamnosus* from normal, intermediate and bacterial vaginosis (BV) microbiota. Data are represented as mean ± SD. ns =not significant and * *p* < 0.05, indicates statistical significance after performing the Kruskal–Wallis test.

We further investigated whether there was a strain-to-strain difference among the major lactobacillus isolates from the eubiotic and dysbiotic vaginal state. Metabolites of *L. jensenii* isolates from healthy samples had significantly higher d-/l-lactic acid ratios as compared to *L. jensenii* isolated from intermediate and BV samples (*p* = 0.04) (Figure 4a), but the sample size is small. Though not statistically significant, metabolites of other major *Lactobacillus* species from healthy samples had higher d-/l-lactic acid ratio than isolates of same spp. from other groups (Figure 4b–f).

### 3.6. Species-Specific Correlation of Lactic Acid

**Figure 5:**
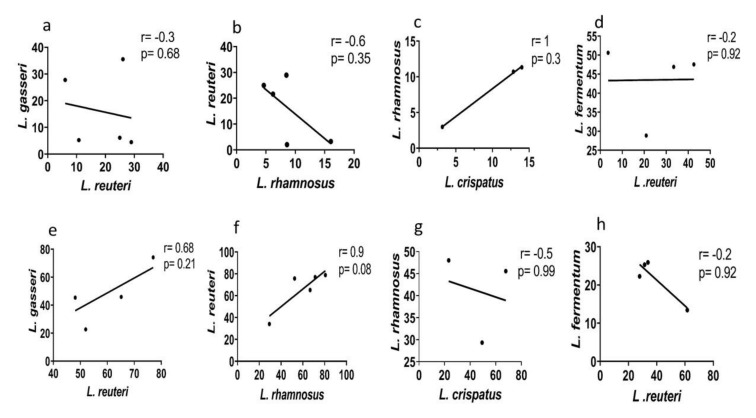
Correlation between *Lactobacillus* species from normal microbiota in d-lactic acid production (a to d- top panel) (**a**) *L. gasseri* with *L. reuteri*, (**b**) *L. reuteri* with *L. rhamnosus*, (**c**) *L. rhamnosus* with *L. crispatus*, (**d**) *L. fermentum* with *L. reuteri* and l- lactic acid production (e to h- bottom panel) of (**e**) *L. gasseri* with *L. reuteri*, (**f**) *L. reuteri* with *L. rhamnosus*, (**g**) *L. rhamnosus* with *L. crispatus*, (**h**) *L. fermentum* with *L. reuteri.* Correlations between species were carried out by Spearman’s rank correlation. The correlation coefficient and *p* value for each analysis are mentioned in the graph.

Normal vaginal microbiota have a heterogeneous lactobacilli population. Our previous studies had shown that majority of normal microbiota harbored at least two *Lactobacillus* species simultaneously [6]. Heterogeneity of lactobacilli population is reduced during dysbiosis (6). To determine the plausible synergistic effect of different *Lactobacillus* species with each other, we did a correlation analysis of lactic acid produced by different *Lactobacillus* isolates from normal microbiota. The concentrations of d-, l- Lactic acid isomers in metabolites of certain *Lactobacillus* species positively correlated with each other while few others negatively correlated (Figure 5a–h).

d-lactic acid produced by *L. rhamnosus* correlated negatively with *L. reuteri* (*r* = −0.6, *p* = 0.35) (Figure 5b) whereas positively with *L. crispatus* (*r* = 1, *p* = 0.3) concentrations of d-lactic acid in CFCs (Figure 5c). l-lactic acid in the CFCs of *L.reuteri* positively correlated with *L.gasseri* and *L.rhamnosus* (Figure 5e,f) and *L. rhamnosus* and *L. crispatus* l-lactic acid amount negatively correlated (Figure 5g).

### 3.7. Hydrogen Peroxide Evaluation

From normal microbiota, 129 (76.33%) isolates, from intermediate 10 (66.67%), and BV microbiota 19 (82.6%) lactobacilli produced H_2_O_2_. To analyze the species-level distribution of H_2_O_2_ producers in different microbiota, intermediate and BV group were merged as dysbiosis group due to smaller of isolates for each species. We observed no statistical difference in the distribution of H_2_O_2_-producing *Lactobacillus* species between the groups (Table 1).

Of the 207 lactobacilli, 158 (76.32%) produced H_2_O_2_ on TMB-HRP MRS medium. At least 80% of isolates of *L. jensenii* (16, 88.89%), *L. salivarius* (12, 85.71%), *L. rhamnosus* (34, 85.0%), *L. vaginalis* (11, 84.62%) and *L. gasseri* (18, 81.82%) produced H_2_O_2_. Semi-quantitative analysis resulted in identification of 95 (45.89%) isolates as strong, 37 (17.87%) lactobacilli as medium and 26 (12.56%) as weak H_2_O_2_ producers. *L. jensenii* (12, 66.67%), *L. rhamnosus* (23, 57.5%), *L. salivarius* (6, 48.86%) and *L. vaginalis* (6, 46.15%) were the major producers of strong H_2_O_2_ (Table 2).

### 3.8. Autoaggregation of Lactobacilli

**Figure 6:**
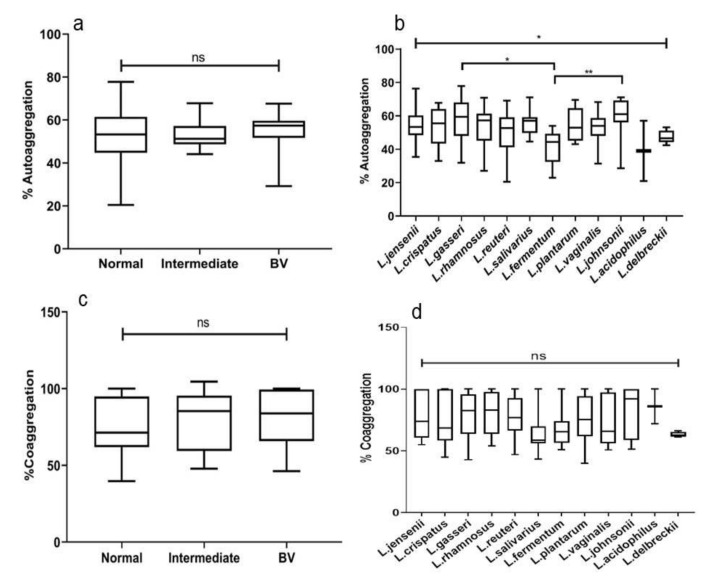
Autoaggregation of (**a**) *Lactobacillus* isolates from different groups and (**b**) species-specific % autoaggregation. *Candida* coaggregation of (**c**) *Lactobacillus* from different microbiota and (**d**) coaggregation of various species. The topmost line indicates the Kruskal–Wallis test and downward pointing lines indicate Dunn’s multiple comparison test. Data are represented as a box plot wherein the box indicates the interquartile ranges, line within the bars represents the median and whiskers represent the minimum and the maximum values. ns = not significant, * *p* < 0.05, ** *p* < 0.01.

Autoaggregating properties of lactobacilli from normal, intermediate, and BV microbiota were similar (*p* = 0.64) (Figure 6a). However significant differences in mean rate of autoaggregation (%) were noted between different species of *Lactobacillus* (*p* = 0.013). *L. johnsonii* (59.3 ± 12), *L. gasseri* (57.4 ± 2.7), exhibited the highest self-aggregation followed by *L. salivarius* (56.1 ± 7.01) and *L. plantarum* (54.9 ± 9.52). % Autoaggregation of *L. fermentum* differed significantly with *L. gasseri* (*p* = 0.01), *L. johnsonii* (*p* = 0.006) (Figure 6b). The self-aggregating property of different strains of the same species from normal, intermediate and BV microbiota did not vary significantly.

### 3.9. Coaggregation of Lactobacillus with C. albicans

About 119 (70.41%), 9 (60%) and 13 (56.5%) lactobacilli from normal, intermediate and BV microbiota, respectively, could agglutinate *C. albicans*. Agglutination of *C. albicans* by lactobacilli was further semi-quantified as strong, medium, and weak based on the size of aggregates (Figure S2). Only 21 (12.42%) lactobacilli demonstrated strong agglutination with *C. albicans*. Of the 21 isolates, 17 (10%), 2 (13.3%), and 2 (8.69%) isolates of normal, intermediate, and BV women showed strong agglutination, respectively.

Furthermore, on quantitation we observed 75.3, 77.3 and 81.8 mean % coaggregation of *C. albicans* by lactobacilli from normal, intermediate, and BV women, respectively. The mean % coaggregation of *C. albicans* with *L. crispatus, L. gasseri, L. reuteri and L. rhamnosus* was 74 ± 20.6, 79.8 ± 17.6, 77.1 ± 15.6 and (80.7 ± 16.6), respectively (Table S1). The % *Candida* coaggregating ability of lactobacilli when evaluated quantitatively were not statistically significant among the groups as well as among the species (Figure 6c,d).

### 3.10. Antagonistic Effect on Pathogens

Growth of pathogens was tested after incubation with lactobacilli metabolites at different time intervals (4 h, 18 h, 24 h). Data represented is of 24 h growth inhibition, since there were no significant differences between readings of 18 h and 24 h. The pathogen inhibitory effects of lactobacilli were similar across the three groups for all the pathogens (Figure S3). Growth of *S. agalactiae* was inhibited up to 30% when grown in its own CFCs.

**Figure 7:**
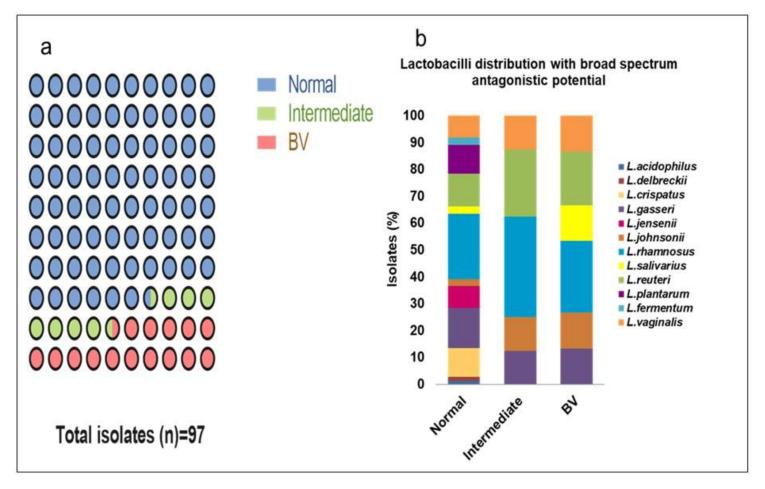
Lactobacilli with broad-spectrum antimicrobial activity. Lactobacilli isolates showing ≥90% growth inhibition on all the tested urogenital pathogens. (**a**) Proportions of lactobacilli isolates from normal, intermediate, and BV microbiota. The parts of the whole plot indicate that 76.29%, 8.25%, 15.46% of the isolates with broad-spectrum antimicrobial activity belonged to normal, intermediate and BV microbiota, respectively. (**b**) *Lactobacillus* species distribution from each normal, intermediate, and BV microbiota. The stack bar graph indicates the diversity of lactobacilli with broad-spectrum antimicrobial potential in normal microbiota is different from intermediate and BV microbiota.

From the 207 lactobacilli, 97 (46.86%) isolates exhibited a broad-spectrum antagonistic effect with at least 90% growth inhibition of all the seven pathogens (Figure 7a). They primarily constitute of *L. rhamnosus* (25, 25%), *L. reuteri* (14, 14%) and *L. gasseri* (14, 14%). From these 97 isolates, 74 (76.29%) belong to normal microbiota whereas 8 (8.25%) and 15 (15.46%) isolates were from intermediate and BV microbiota, respectively. *L. rhamnosus* (18, 24.32%) and *L. gasseri* (11, 14.86%) were the predominant species in normal microbiota whereas *L. rhamnosus* (3, 37.5%), (4, 26.67%) and *L. reuteri* (2, 25%), (3, 20%) were the prevalent lactobacilli in intermediate and BV microbiota with broad-spectrum antimicrobial activity (Figure 7b).

Furthermore, *L. crispatus* (8, 8.23%), *L. plantarum* (8, 8.23%), *L. jensenii* (6, 6.19%), *L. fermentum* (2, 2.06%), *L. acidophilus* (1, 1.03%), and *L. delbrueckii* (1, 1.03%) also demonstrated a broad range of antimicrobial action that was present only in normal microbiota. These six species from normal microbiota constituted 26.80% (26/97) of the isolates with a broad range of antimicrobial properties.

### 3.11. Association of Probiotic Properties from Different Microbiota

Hierarchical clustering analysis showed different clustering of patterns of lactic acid in normal microbiota compared to intermediate and BV groups (Figure S4a–c). In BV microbiota, d-lactic acid clustered separately from total lactic acid and l-lactic acid (Figure S4c).

**Figure 8:**
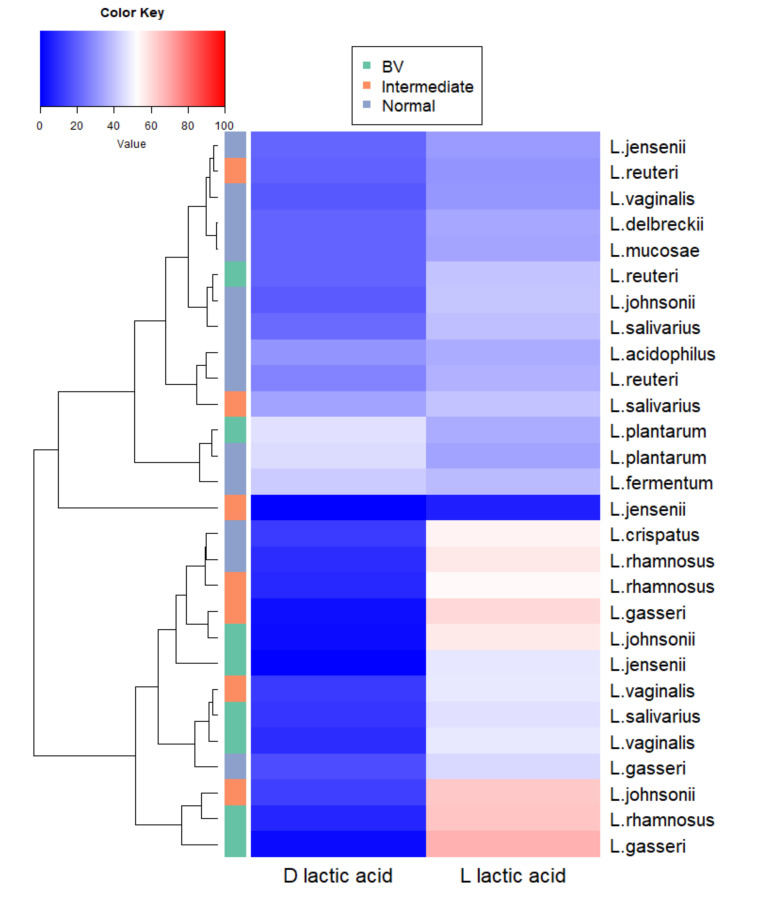
Heatmap of concentrations of lactic acid produced by different species of *Lactobacillus* present in normal, intermediate and asymptomatic BV microbiota. Mean values of d- and l-lactic acid amount in CFCs of each species from the three groups were used for hierarchical clustering. *Lactobacillus* species are shown on the right side and the microbiota from which they were isolated are represented on the left side of the heatmap. A color bar with scales is shown, indicating that dark red corresponds to the maximum value and dark blue to minimum value.

We further performed hierarchical clustering of lactic acid isomers produced by *Lactobacillus* species from normal, intermediate and BV microbiota. We could see the majority of lactobacilli species from intermediate and asymptomatic BV clustered together rather than with the species from normal microbiota (Figure 8).

*L. jensenii*, *L. johnsonii* from normal microbiota clustered distinctly from *L. jensenii*, *L. johnsonii*, respectively, from intermediate and asymptomatic BV microbiota (Figure 8).

**Figure 9:**
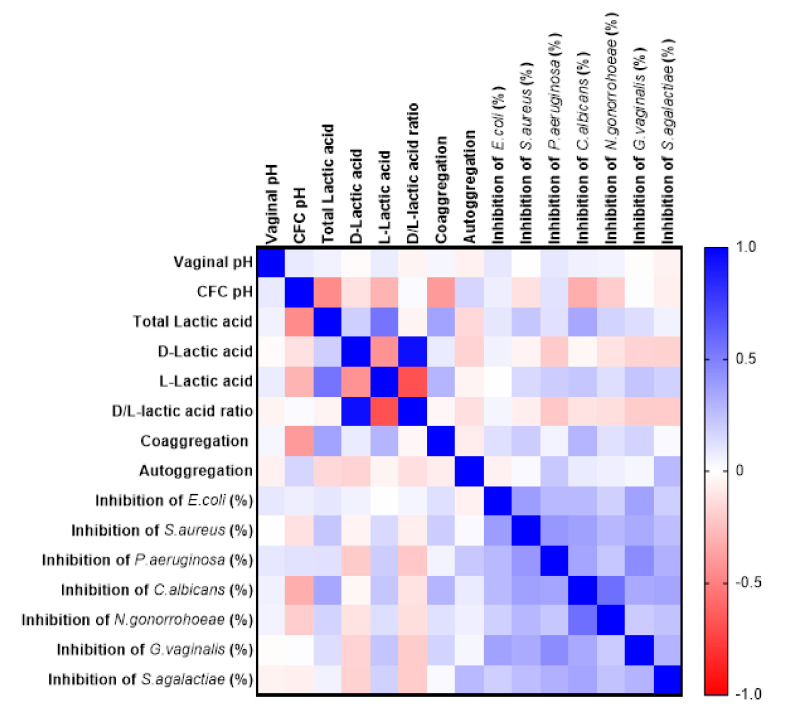
Relationship of different functional properties of lactobacilli with each other. The Spearman’s correlation matrix indicates the correlation nature between the lactobacilli probiotic traits. Dark blue color on the scale denotes a perfect positive correlation (*r* = 1) and dark red indicates a perfect negative correlation (*r* = −1).

Inhibition of the pathogens was positively correlated to pH and lactic acid content of metabolites. The inhibitory effects on *S. aureus*, *C. albicans* and *N. gonorrhoeae* were positively correlated to total and l-lactic acid present in the CFCs. Inhibition of the pathogens positively correlated to the l-lactic acid amount (Figure 9). Coaggregating and autoaggregating ability of lactobacilli isolates positively correlated with the growth inhibition of *C. albicans*.

## 4. Discussion

The efficacy of asymptomatic BV treatment with antibiotics is often debatable [7,24]. With no risk of antimicrobial resistance, the use of probiotic lactobacilli as a prophylaxis for symptomatic BV appears coherent. Asymptomatic BV is also highly prevalent in women, associated with a lactobacilli-deficient condition [6,25] and an increased risk of infection due to disruption of the vaginal epithelium [26]. However, it is largely unknown whether the lactobacilli present during eubiosis have different functional properties than lactobacilli during asymptomatic BV. To decipher the probiotic properties of 207 lactobacilli from contrasting vaginal niche, we examined their antimicrobial metabolites and antagonistic potential towards various urogenital pathogens.

Acidification is the primary mechanism by which *Lactobacillus* protects the vaginal microenvironment from pathogenic bacteria [13,27]. In this study, the total lactic acid in lactobacilli metabolites did not vary significantly between the groups. These observations are in contrast to previous reports on lactic acid from eubiotic and dysbiotic microbiota [12,28]. This discrepancy could be due to the detection of lactic acid from cervicovaginal fluid samples in the earlier reports and the use of axenic cultures in the present study. This implies that during asymptomatic BV, which is characterized by reduction in *Lactobacillus* abundance and diversity, the remaining lactobacilli strains are capable of producing lactic acid when suitable environmental conditions are provided. d-lactic acid, which is an isomer of l-lactic acid, has a greater protective role than l- lactic acid [13]. We report remarkably higher levels of d-LA and high d/l lactic acid ratios in metabolites of lactobacilli from normal microbiota compared to those from asymptomatic BV. d-lactic acid is exclusively contributed by the bacteria, whereas l- lactate is produced both by the bacteria and vaginal epithelial cells [29]. Recently higher d-lactic acid was reported in axenic cultures and cervicovaginal mucus samples from women with normal microbiota [30,31]. Thus, presence of d-lactic acid producing lactobacilli in the vaginal milieu is more important than any lactic acid producing species. We found isolates of *L. fermentum, L. plantarum, L. reuteri* were highest d-LA producers and *L. rhamnosus*, *L. crispatus*, *L. johnsonii* were the highest l-LA producers and. Furthermore, *L. jensenii* isolates from normal microbiota produced higher d-lactic acid than *L. jensenii* from intermediate and asymptomatic BV. Studies have associated *L.jensenii* with normal microbiota [32,33] and presence of d-lactic acid producing *L. jensenii* suggest protective effect of the species to maintain vaginal homeostasis. Thus, though lactobacilli strains from asymptomatic BV could acidify the medium and produce total lactic acid comparable to lactobacilli from normal microbiota, they could not produce an equivalent amount of d-lactic acid like their counterparts from normal microbiota.

In healthy vaginal microbiota, which is characterized by a heterogeneous *Lactobacillus* population, the protective outcome of these lactobacilli could be the result of their synergistic effects [6]. We noted certain lactobacilli species positively correlated in lactic acid and its isomer production. Additionally, the correlation of lactic acid concentrations produced by *L. gasseri* and *L. reuteri* strains from normal microbiota differed from dysbiotic microbiota. This difference between normal and dysbiotic microbiota in lactobacilli co-species relation indicates the presence of different strains of species. Thus, the interaction and relation among the *Lactobacillus* species may differ in a healthy environment and perturbed condition like BV. It will be interesting to note the composition of the metabolites when co-cultured. Such synergistic efficacy among the species forms the basis of selection of a multistrain consortia instead of a single probiotic strain to restore vaginal homeostasis.

Hydrogen peroxide is another metabolite of lactobacilli reported to have antimicrobial potential and immunomodulatory effect [33]. Studies have associated the presence of H_2_O_2_-producing lactobacilli with normal microbiota [34,35]. We did a semi-quantitative evaluation of H_2_O_2_ on the MRS-TMB-HRP medium, due to its instability in CFCs. We observed no significant difference in H_2_O_2_-producing lactobacilli between normal and dysbiotic microbiota. We did a qualitative or semi-quantitative estimation of H_2_O_2_ from the lactobacilli on solid medium. However, a quantitative estimation of hydrogen peroxide in liquid medium probably would confirm whether there was any difference in the production of H_2_O_2_ by lactobacilli isolated from normal, intermediate and BV microbiota.

Adherence to vaginal epithelial cells is another mechanism by which lactobacilli can colonize and sustain in the vaginal microenvironment. Autoaggregation of lactobacilli is an indicator of adherence ability and biofilm formation on host mucosa [36,37]. We observed species-specific lactobacilli self-aggregation and no significant difference among the microbiota groups. This observation was not surprising, because strains with poor adherence properties will not be able to colonize and will be lost from the vaginal microbiota and not recovered from the vaginal samples. Isolates of *L. crispatus, L. fermentum, L. acidophilus, and L. delbrueckii* were not present in women with asymptomatic BV [6]. A compromised adhering strength could be one of the plausible reasons for their absence from asymptomatic BV. Among the species that were recovered, isolates of *L. gasseri* and *L. johnsonii* had better autoaggregating abilities than isolates of *L. fermentum*. Moreover, *L. johnsonii, L. gasseri*, and *L. salivarius* displayed better autoaggregation than other species.

Coaggregation of lactobacilli with pathogens results in the physical elimination of pathogens (Pino, 2019). In this study, isolates of *L. acidophilus, L. johnsonii* and *L. rhamnosus* demonstrated the commendable coaggregating strength of *C. albicans*. Earlier *L. crispatus* was reported to have the highest *C. albicans* aggregating property [38]. Similar to autoaggregation, the *C. albicans* coaggregating property of lactobacilli did not vary among the groups. Desirable probiotic lactobacilli should have a strong adhesive strength for efficient pathogen displacement [18]. *C. albicans* is the main causative agent for vulvovaginal candidiasis [39], which is the second most gynecological infection of women in reproductive age [40]. Exploring these *Candida* coagglutinating lactobacilli strains seems a promising probiotic strategy against vulvovaginal candidiasis.

We further tested the antimicrobial effects of vaginal lactobacilli from normal and dysbiotic microbiota on the major urogenital pathogens causing aerobic vaginitis and urinary tract infections (*E. coli, S. aureus, P. aeruginosa*), bacterial vaginosis (*G. vaginalis*), vulvovaginal candidiasis (*C. albicans*), gonorrhea (*N. gonorrhoeae*) and preterm birth (*S. agalactiae*) [41]. Studies have reported lactic acid as the key lactobacilli defense factor against various pathogens [12,28,42]. Since total lactic acid did not vary between lactobacilli from normal and dysbiotic microbiota, their antagonistic activity might have remained similar.

Nearly half of the isolates exhibited broad-spectrum (>90% inhibition) antimicrobial activity and about three quarters of these lactobacilli belonging to normal microbiota predominantly consisted of *L. rhamnosus, L. reuteri* and *L. gasseri*. Recently, *L. rhamnosus* from vaginal samples have been reported to demonstrate broad-spectrum antagonistic activity [43]. Different studies have demonstrated the antimicrobial effect of selected lactobacilli on some pathogens [44,45]. However, an extensive evaluation of the probiotic properties of such a large number of lactobacilli was not reported earlier.

We observed a positive correlation of lactobacilli and *C. albicans* coaggregation with inhibition of *C. albicans* by lactobacilli metabolites. Besides lactic acid, metabolites of lactobacilli contain exopolysachharides (EPS) and membrane vesicles (MVs). Both EPS and MVs from lactobacilli can affect the adhesion of *C. albicans* on host epithelial cells [46]. Exoploysachharides present in lactobacilli metabolites have been reported to affect the growth of *C. albicans* by extending its lag phase. Thus, lactobacilli with higher *C. albicans* coaggregating ability may have a better growth inhibitory effect on *C. albicans* due to the presence of exopolysachharides.

Since lactic acid isomers were the quantitative variable that differed between the groups, we further analyzed the clustering pattern of the species. The species picked from intermediate and asymptomatic BV clustered together and were separated from the species from normal microbiota. This indicates that, though lactobacilli from asymptomatic BV may be similar to lactobacilli from normal microbiota in certain traits, they are different in some properties at the species and strain level.

Our study is limited by the fact that we have not evaluated other functional attributes of *Lactobacillus* such as other organic acids, bacteriocin, biofilm formation, or coaggregation with other pathogens. Despite being the prevalent vaginal lactobacilli, we were unable to evaluate *L. iners* due to its inability to grow on MRS [6]. In spite of these shortcomings, we could depict a broad view of the distinct functional traits of vaginal lactobacilli from normal, intermediate and asymptomatic BV microbiota which was not reported earlier with such an extensive sample size. It will be interesting to note the behavior of these lactobacilli along with other microbial co-members to further decipher their potential. Additionally, other functional traits such as their anticancer effects needs to be explored. The meritorious shortlisted lactobacilli will be evaluated for these traits in the future.

## 5. Conclusions

Numerous studies have reported the diversity of vaginal microbiota, but the functional aspects of lactobacilli, especially in asymptomatic BV, was overlooked. Our findings suggest that asymptomatic BV is characterized by the absence of beneficial strains of lactobacilli and should not be left untreated. A carefully selected microbial consortium of lactobacilli should be considered for the treatment of asymptomatic cases of BV to prevent any future episodes of other RTIs and STIs.

## Figures and Tables

**Table 1:** Diversity of H_2_O_2_ production by *Lactobacillus* species from normal and dysbiotic (intermediate and BV) microbiota. Semi-quantitative measurement of H_2_O_2_ production on MRS-TMB-HRP agar by lactobacilli isolates was evaluated based on the intensity of blue color obtained on colonies. *n* = number of isolates of *Lactobacillus* species. Fisher’s exact test was carried out to determine the statistical significance.

*Lactobacillus* Species	H_2_O_2_ Production	Normal	Dysbiosis	*p* Value
*L. crispatus* (*n* = 14)	H_2_O_2_ producer	10	0	_
	H_2_O_2_ non producer	4	0	
*L. gasseri* (*n* = 22)	H_2_O_2_ producer	15	3	0.99
	H_2_O_2_ non producer	4	0	
*L. jensenii* (*n* = 18)	H_2_O_2_ producer	15	1	0.22
	H_2_O_2_ non producer	1	1	
*L. johnsonii* (*n* = 11)	H_2_O_2_ producer	4	3	0.99
	H_2_O_2_ non producer	3	1	
*L. rhamnosus* (*n* = 40)	H_2_O_2_ producer	23	11	0.64
	H_2_O_2_ non producer	3	3	
*L. salivarius* (*n* = 14)	H_2_O_2_ producer	9	3	0.99
	H_2_O_2_ non producer	2	0	
*L. reuteri* (*n* = 42)	H_2_O_2_ producer	26	5	0.99
	H_2_O_2_ non producer	9	2	
*L. plantarum* (*n* = 13)	H_2_O_2_ producer	6	1	0.99
	H_2_O_2_ non producer	5	1	
*L. fermentum* (*n* = 11)	H_2_O_2_ producer	7	0	_
	H_2_O_2_ non producer	4	0	
*L. vaginalis* (*n* = 13)	H_2_O_2_ producer	9	2	0.42
	H_2_O_2_ non producer	1	1	

**Table 2:** Semi-quantitative distribution of H_2_O_2_ production by different *Lactobacillus* species. The number in the parenthesis denotes the percentage.

*Lactobacillus* Species	Number of H_2_O_2_ Non Producers	Number of H_2_O_2_ Producers			
		Weak	Medium	Strong	Total
*L. acidophilus* (*n* = 2)	1(50)	0 (0)	1 (50)	0 (0)	1 (100)
*L. delbrueckii* (*n* = 5)	2 (40.0)	0 (0)	1 (20.0)	2 (40.0)	3 (60.0)
*L. crispatus* (*n* = 14)	4 (28.57)	0 (0)	5 (35.71)	5 (35.71)	10 (71.43)
*L. gasseri* (*n* = 22)	4 (18.18)	4 (18.18)	5 (22.72)	9 (40.91)	18(81.82)
*L. jensenii* (*n* = 18)	2 (11.11)	1 (5.56)	3 (16.67)	12 (66.67)	16 (88.89
*L. johnsonii* (*n* = 11)	4 (36.36)	2 (18.18)	2 (18.18)	3 (27.27)	7 (63.63)
*L. rhamnosus* (*n* = 40)	6 (15.0)	5 (12.5)	6 (15)	23 (57.5)	34 (85.0)
*L. salivarius* (*n* = 14)	2 (14.29)	2 (14.29)	4 (28.57)	6 (48.86)	12 (85.71)
*L. reuteri* (*n* = 42)	11 (26.19)	7 (16.67)	6 (14.29)	18 (42.86)	31 (73.81)
*L. plantarum* (*n* = 13)	6 (46.15)	2 (15.38)	0 (0)	5 (38.46)	7 (53.85)
*L. fermentum* (*n* = 11)	4 (36.36)	1 (9.09)	1 (9.09)	5 (45.45)	7 (63.63)
*L. vaginalis* (*n* = 13)	2 (15.38)	2 (15.38)	3 (23.08)	6 (46.15)	11 (84.62)

## Supplementary Materials

The following are available online at https://www.mdpi.com/2076-2607/8/12/1949/s1, Figure S1: pH of MRS medium after growth of major *Lactobacillus* species (a) *L. jensenii* (b) *L. gasseri* (c) *L. reuteri* (d) *L. salivarius* (e) *L. johnsonii* (f) *L. rhamnosus* from normal, intermediate and BV microbiota, Figure S2: Macroscopic (slide) and microscopic (under 10× and 40× magnification) view of *Lactobacillus* isolates Co-aggregation with *C. albicans*. (a) No agglutination showing no macroscopic aggregates (b) weak agglutination having small aggregates with visible clusters of bacteria (c) medium agglutination showing larger aggregates (d) Strong agglutination having macroscopically larger clumps, Figure S3: Antimicrobial effect of lactobacilli CFCs from different microbiota on (a) *E. coli* (b) *S. aureus* (c) *P. aeruginosa* (d) *C. albicans* (e) *G. vaginalis* (f) *N. gonorrhoeae* (g) *S. agalactiae* after 24 h incubation, Figure S4. Heatmap comparison of lactobacilli functions. Functional properties of each *Lactobacillus* isolate (*n* = 207) from (a) normal (b) intermediate and (c) BV microbiota are represented, Table S1: Functional properties of different species of *Lactobacillus* used in the study. Kruskal-Wallis test was used to determine statistical significance.
