# Supplementary material for: Distinct Functional Traits of Lactobacilli from Women with Asymptomatic Bacterial Vaginosis and Normal Microbiota
Source: Microorganisms. 2020 Dec 9;8(12):1949. doi: 10.3390/microorganisms8121949 (PMC7763271; doi:10.3390/microorganisms8121949)
Supplement: Supplementary file 1 [file microorganisms-08-01949-s001.pdf]

| | n (%) | CFC pH Mean ±SD | Lactic acid (mM) Mean ±SD | D-LA (mM) Mean±SD | L-LA (mM) Mean±SD | D/L ratio Mean±SD | Coaggregation (%) Mean±SD | Autoaggregation (%) Mean±SD |
|---|---|---|---|---|---|---|---|---|
| *L.acidophilus* | 2(0.96) | 4.5±0.71 | 62.39±21.21 | 31.02±18.90 | 35.21±6.88 | 0.84±0.37 | 86±19.8 | 38.9±25.5 |
| *L.delbreuckii* | 5(2.4) | 4.4±1.08 | 57.59±18.49 | 20.55±12.87 | 34.73±20.91 | 1.13±1.19 | 63.18±2.07 | 47.49±3.98 |
| *L.crispatus* | 14(6.76) | 4.29±0.64 | 63.32±16.07 | 11.65±11.91 | 53.93±18.84 | 0.29±0.47 | 74±20.6 | 53.3±11.7 |
| *L.gasseri* | 22(10.62) | 4.2±0.75 | 56.62±14.22 | 14.15±16.52 | 46.09±18.88 | 0.41±0.50 | 79.8±17.6 | 57.4±12.7 |
| *L.jensenii* | 18(8.69) | 4.22±0.58 | 49.06±16.27 | 18.72±15.33 | 31.76±16.02 | 0.91±1.4 | 76.4±17.4 | 54.2±9.04 |
| *L.johnsonii* | 11(5.31) | 3.95±0.68 | 69.80±18.39 | 13.85±15.17 | 47.33±21.62 | 0.45±0.59 | 83.40±20 | 59.3±12 |
| *L.rhamnosus* | 40(19.32) | 3.96±0.65 | 68.77±17.26 | 8.96±8.19 | 57.61±18.49 | 0.20±0.25 | 80.7±16.6 | 53.3±10.4 |
| *L.salivarius* | 14(6.76) | 4.11±0.45 | 61.15±10.94 | 19.76±14.10 | 41.19±18.91 | 0.64±0.58 | 64.2±16.3 | 56.1±7.01 |
| *L.reuteri* | 42(20.29) | 4.2±0.73 | 62.55±18.47 | 26.39±18.17 | 36.93±18.41 | 0.95±0.81 | 77.1±15.6 | 49.4±13.3 |
| *L.plantarum* | 13(6.28) | 3.77±0.70 | 74.11±15.72 | 42.19±23.45 | 35.00±17.80 | 1.70±1.31 | 74.1±19.1 | 54.9±9.52 |
| *L.fermentum* | 11(5.31) | 3.92±0.41 | 67.58±17.03 | 38.09±21.71 | 39.19±25.51 | 1.35±0.97 | 69.64±16.71 | 41.81±9.53 |
| *L.vaginalis* | 13(6.28) | 4.42±0.57 | 57.17±16.29 | 16.56±19.71 | 35.16±10.38 | 0.57±0.76 | 72.1±19.3 | 53.7±9.74 |
| *Lactobacillus sps.* | 2(0.96) | 4.75±0.35 | 61.28±3.70 | 14.24±8.27 | 41.62±10.40 | 0.38±0.31 | 80.93±26.96 | 47.42±3.04 |
| P value | - | 0.003 | 0.166 | <0.0001 | 0.012 | <0.0001 | 0.132 | 0.013 |

Supplementary Table 1: Functional properties of different species of *Lactobacillus* used in the study. Kruskal-Wallis test was used to determine statistical significance

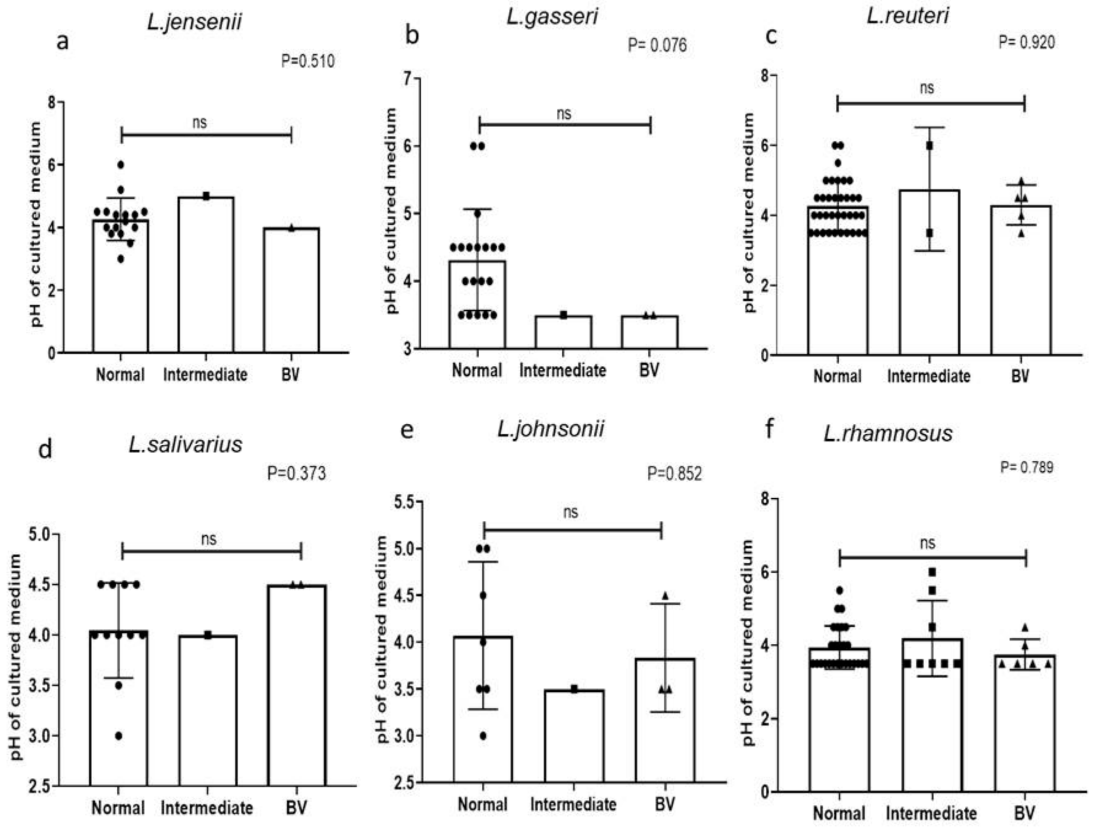


Figure S1: pH of MRS medium after growth of major *Lactobacillus* species (a) *L.jensenii* (b) *L.gasseri* (c) *L.reuteri* (d) *L.salivarius* (e) *L.johnsonii* (f) *L.rhamnosus* from normal, intermediate and BV microbiota. Data are represented as mean ± SD. ns =not significant and *P<0.05, indicates statistical significance after performing the Kruskal-Wallis test.

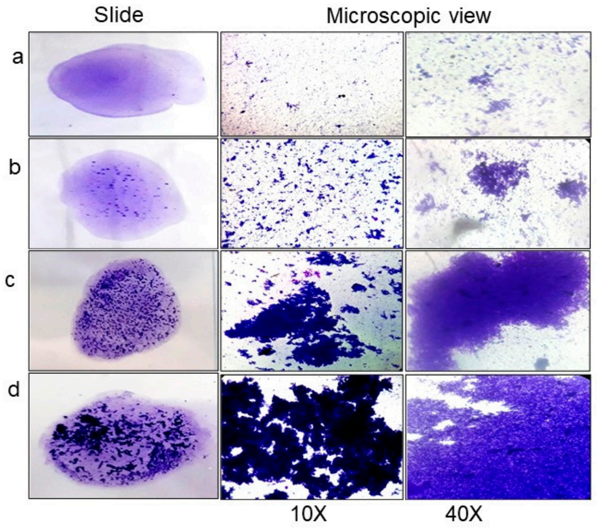


Figure S2: Macroscopic (slide) and microscopic (under 10X and 40X magnification) view of *Lactobacillus* isolates Co-aggregation with *C.albicans*. a) No agglutination showing no macroscopic aggregates b) weak agglutination having small aggregates with visible clusters of bacteria c) medium agglutination showing larger aggregates d) Strong agglutination having macroscopically larger clumps

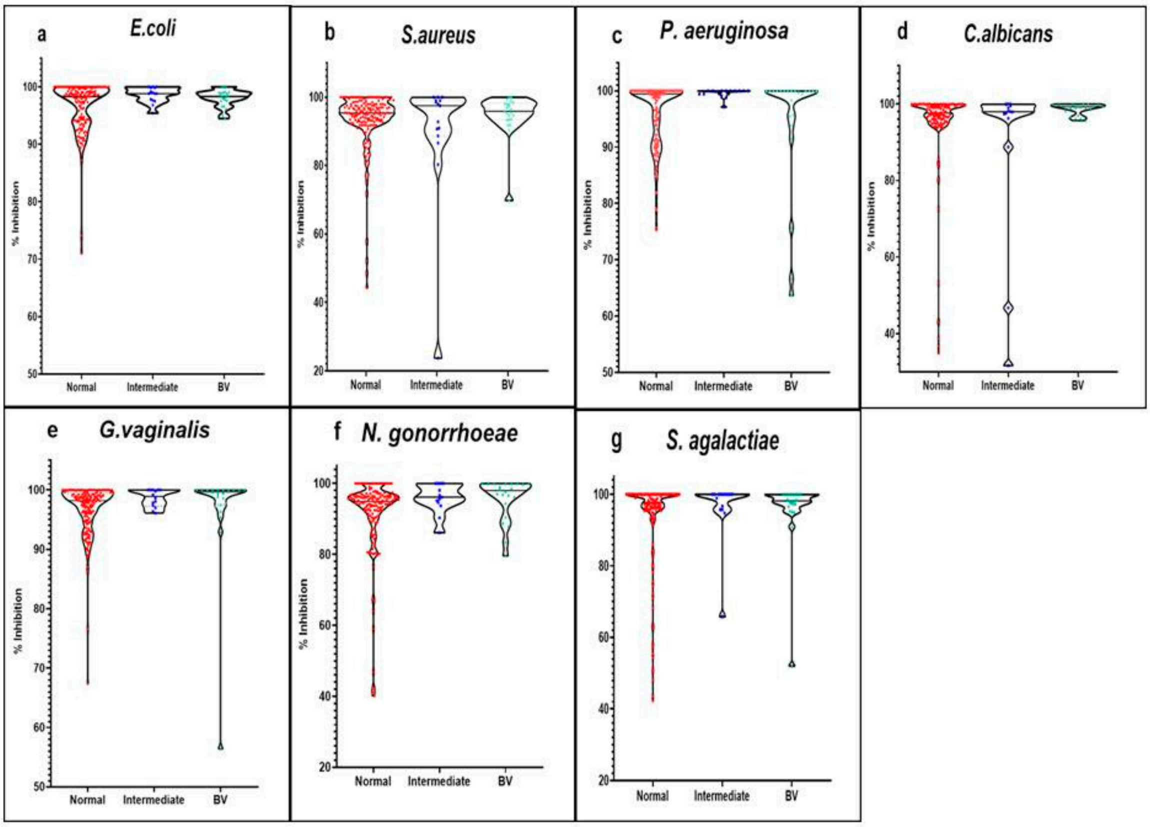


Figure S3: Antimicrobial effect of lactobacilli CFCs from different microbiota on a) *E. coli* b) *S. aureus* c) *P. aeruginosa* d) *C. albicans* e) *G. vaginalis* f) *N. gonorrheae* g) *S.agalactiae* after 24h incubation.

Data for each isolate is expressed as mean % inhibition of growth. Differences between the medians of each group was calculated by the Kruskal-Wallis test (P=0.212).

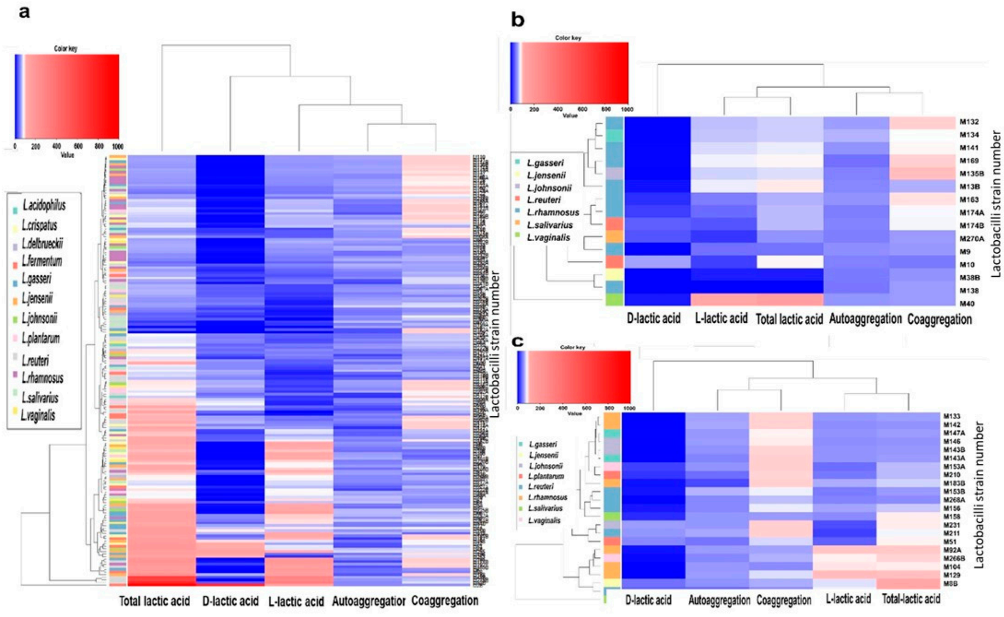


**Figure S4.** Heatmap comparison of lactobacilli functions. Functional properties of each *Lactobacillus* isolate (n=207) from a) normal b) intermediate and c) BV microbiota are represented. The clustering differentiates the functional profile of lactobacilli isolates from normal microbiota to lactobacilli from intermediate and BV microbiota. A color bar with scales for each heatmap is included, indicating that dark-red corresponds to the maximum value and dark-blue to minimum value.
